# Decoding global precipitation processes and particle evolution using unsupervised learning

**DOI:** 10.1126/sciadv.adu0162

**Published:** 2025-09-19

**Authors:** Fraser King, Claire Pettersen, Brenda Dolan, Julia Shates, Derek Posselt

**Affiliations:** ^1^ Climate and Space Sciences and Engineering, University of Michigan, Ann Arbor, MI, USA.; ^2^ Colorado State University, Fort Collins, CO, USA.; ^3^ Jet Propulsion Laboratory, California Institute of Technology, Pasadena, CA, USA.

## Abstract

High-quality hydrometeor microphysical observations are essential for accurate precipitation estimates and for evaluating weather and climate models. However, analyzing these properties is challenging due to their high variability, complex interactions, and large data volumes. In this study, we examine more than 1.5 million minute-scale rain and snow particle attributes and collocated meteorological variables from seven global measurement sites over 9 years. Applying Uniform Manifold Approximation and Projection (UMAP) for nonlinear dimensionality reduction, we reduce the dataset’s dimensionality by 75%, identifying nine distinct precipitation groups and associated particle evolution pathways. UMAP effectively captures the global structure of precipitation phases such as rain, snow, and mixed-phase types, revealing clear patterns that linear methods struggle to resolve. The resulting UMAP manifold offers a unique perspective on precipitation phase and intensity, advancing our understanding of particle evolutionary processes and offering valuable insights for improving weather and climate models and remote sensing precipitation estimates.

## Teaser

Analyzing rain and snow particles reveals hidden patterns, improving precipitation classification for better model predictions.

## INTRODUCTION

Climate change has substantially altered global precipitation patterns, increasing the variability and intensity of precipitation events (*1*–*4*). The rise in extreme precipitation is closely linked to hazards such as flooding, infrastructure damage, and degradation of water quality from surface runoff (*5*–*7*). Warming temperatures also affect regional distributions of precipitation phase, including the frequency of rain, snow, and mixed-phase (liquid and solid) particles (*8*, *9*). Accurately determining whether precipitation falls as rain or snow is critical for hydrologic and climate modeling, as it governs snowpack accumulation, runoff timing, and freshwater availability (*10*). However, observations of particle phase are traditionally challenging to obtain due to the sparse coverage of in situ measurement sites, the scarcity of airborne probe data, and the uncertainty in satellite partitioning algorithms, particularly over high latitudes (*11*, *12*). These challenges lead to structural and parametric errors in microphysical representations of precipitation phase in weather and climate models, which contributes substantial error in their predictions of radiation and cloud features (*13*, *14*). Robust precipitation-phase partitioning methods are therefore critically important for reliable predictions of surface properties, particularity in regions that commonly experience temperatures near 0°C (*15*).

Observationally, many weather stations and in situ phase partitioning algorithms rely on surface air temperature as a direct proxy for precipitation type, an approach that becomes unreliable in the critical −3° to +5°C range where rain and snow occur with nearly equal frequency (*16*, *17*). In this transition zone, even small errors in measured temperature or humidity can lead to misclassification, and mixed-phase events (e.g., sleet, freezing rain, or rain-snow mixtures) are notoriously difficult to identify due to their complex microphysical characteristics (*18*). Numerical models face similar challenges where simplified phase partitioning schemes (e.g., using a fixed 0°C cutoff) often misrepresent reality, introducing biases in simulated snowfall resulting in errors of tens of centimeters in snow water equivalent and substantial shifts in the timing of peak streamflow (*15*). While more sophisticated schemes that incorporate humidity or vertical temperature profiles (such as wet-bulb-temperature–based parameterizations) can improve overall skill, they do not generalize effectively across all climate regimes, particularly in areas with highly complex precipitation dynamics (*10*, *19*). These challenges underscore the critical need for large-scale, global datasets that better capture the complexity of precipitation processes and associated meteorological conditions to facilitate the development of more nuanced and regionally adaptive parameterizations that can accurately simulate phase transitions.

To monitor precipitation globally, we increasingly rely on spaceborne remote sensing techniques, which provide critical insights into the motion and spatial extent of precipitating cloud systems at high spatiotemporal resolution (*20*–*22*). Spaceborne systems provide valuable observations over vast, remote regions that would be expensive to monitor using traditional ground or aircraft-based in situ instruments [e.g., (*23*–*26*)]. However, the algorithms used to estimate precipitation from satellite observations depend on the microscale atmospheric processes that influence cloud physics and falling hydrometeors (*27*–*29*). Approximations necessary to simplify the largely unobservable microscale complexity of cloud processes contribute substantial uncertainty to satellite-based estimates of precipitation (*30*, *31*). Moreover, cloud microscale (hereafter microphysical) processes constitute one of the dominant sources of uncertainty and error in weather and climate prediction models. Collectively, these observational, modeling, and remote-sensing limitations are most acute across the mid- and high-latitudes, where slight temperature variations around 0°C can substantially affect the accuracy of the estimated surface precipitation phase and rate.

Minimizing these uncertainties is crucial, but doing so requires knowledge of the environmental dependence of cloud microphysical properties. Obtaining robust patterns from particle microphysical observations is challenging due to the vast number of particles present in clouds; the large variety of ice particle shapes and sizes; and the complex, nonlinear interactions between cloud microphysical and dynamical processes (*32*–*34*). Dimensionality reduction is an effective technique for simplifying complex, multidimensional datasets to reveal underlying latent features (*35*–*37*). For precipitation, dimensionality reduction can reveal key precipitation characteristics across different regional climates, adding valuable context about the atmospheric state at the time of observation (*38*–*40*). This work examines both linear and nonlinear dimensionality reduction techniques, including principal components analysis (PCA) (*41*), and Uniform Manifold Approximation and Projection (UMAP) (*42*), to identify latent embedded features in a large, multidimensional precipitation dataset from the precipitation imaging package (PIP) (*43*). The PIP dataset contains comprehensive measurements of particle size distributions (PSDs), fall speeds, densities, and total particle counts, along with collocated surface conditions from nearby weather stations (e.g., temperature, humidity, pressure, and wind speed). These properties display complex nonlinear interdependencies that are critical for understanding the dynamic processes governing precipitation formation and particle evolution across diverse regional climates.

Previous literature from (*44*) and (*45*) demonstrated that PCA is effective at identifying physically meaningful patterns in both rainfall and snowfall data when analyzed independently. However, the nonlinear relationships between precipitation variables often cause many cases to cluster at the origin, making it difficult to separate them into distinct, physically meaningful groups. Unlike PCA, UMAP reduces the dimensionality of the dataset without assuming that relationships between variables are linear. It achieves this by creating a low-dimensional manifold representation of the dataset that preserves local and global relationships between variables (*42*, *46*, *47*). Previous studies have effectively leveraged information from UMAP-derived embeddings to uncover meaningful patterns in large datasets, further validating its utility in revealing complex features beyond simple visualization (*48*–*52*). By examining the structure of the UMAP coordinate space, we can learn what the latent dimensions represent physically, to extract robust features that describe the underlying structure of regional precipitation.

In this work, we use UMAP to reduce a 12-dimensional (12D) dataset of 128,233 5-min aggregated observations from a specialized high-speed video camera (hereafter called a disdrometer) of rain, snow, mixed-phase precipitation, and nearby surface meteorological observations to a 3D manifold. We first analyze the UMAP coordinate space and associated clusters of observed cases to understand the general structure of precipitation encoded within the manifold. Next, we compare the coordinate spaces generated by UMAP and PCA to determine whether UMAP’s nonlinear dimensionality reduction provides a clearer representation of the precipitation processes than the embeddings produced by PCA. We then examine the number of ambiguous cases identified by each method to assess differences in each dimensionality reduction technique’s global structure. Last, we compare the precipitation groups derived from UMAP with collocated vertically pointing radar measurements to evaluate their physical consistency with independent observations. Ultimately, this work lays the foundation for enhanced precipitation retrievals and refined model parameterizations and describes a pathway toward a deeper understanding of the global water-energy budget. A summary overview of the data, methodology, and operational applications is provided in Materials and Methods. Additional information regarding the input dataset, preprocessing steps, and dimensionality reduction techniques is provided in the Materials and Methods section.

## RESULTS

### Nonlinear precipitation clusters

**Fig. 1.**
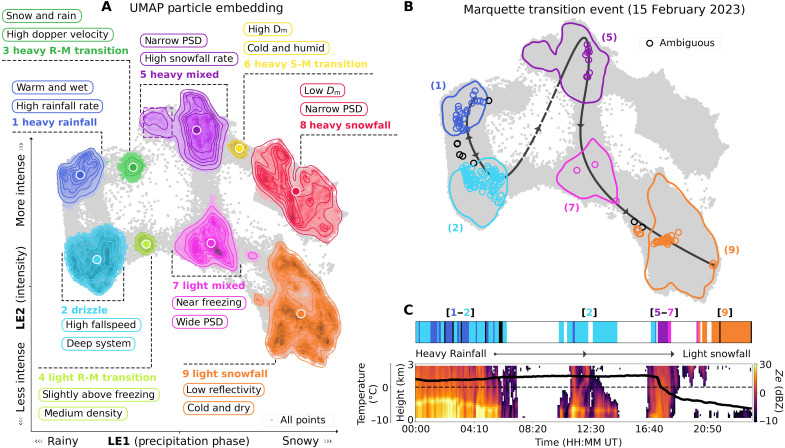
Overview of precipitation clusters derived from UMAP and HDBSCAN. (**A**) UMAP coordinate space for all observations across all sites (gray points), overlaid with colored HDBSCAN-derived density clusters represented as KDE contours (centroids shown as white circles), and annotated with attributed physical precipitation types and key summary characteristics. Coordinates are plotted in the UMAP embedding space for precipitation phase (LE1) and storm intensity (LE2). A 3D visualization with particle shape (LE3) included is shown in Fig. 2. The ambiguous group is not separately highlighted. S stands for snowfall; M, stands for mixed; and R stands for rain. (**B**) Example day at the Marquette (MQT), Michigan site on 15 February 2023, showing discrete 5-min interval UMAP coordinates colored by precipitation groups from (A), with ambiguous points in black (all observations in gray). The directional line indicates the path of time throughout the day, highlighting the evolution of precipitating particles in response to changing local weather conditions (from heavy rain to partially frozen mixed-phase particles to light snowflakes by the end of the day). This transition pathway is shown alongside independent, collocated radar reflectivity measurements in (**C**) overlaid with 2-m air temperature observations in black (dashed line indicating 0°C), highlighting a rapid cooling event at 17:00 UT that aligns with the shift from rain to mixed-phase precipitation and lastly leading to low-intensity snowfall as temperatures continue to decrease until 24:00 UT.

UMAP reduced the dimensionality of the input dataset from 12 dimensions to 3, yielding physically meaningful latent embeddings. Similar to the derived empirical orthogonal functions (EOFs) from a PCA, the UMAP latent embeddings (LE1, LE2, and LE3) were found to encode critical information related to precipitation phase, storm intensity, and particle size/shape, respectively. An analysis exploring higher latent dimensions (e.g., four and five dimensions) revealed that deeper embeddings lacked clear separation and physical significance compared to results using three latent dimensions. While UMAP reduces the data to a 3D coordinate space, it does not cluster points together. By applying hierarchical density-based spatial clustering of applications with noise (HDBSCAN) to this embedding, we can group nearby points, revealing nine distinct physical precipitation clusters. These hierarchical clusters are primarily separated in the first two embedding spaces (LE1 and LE2), representing precipitation phase and intensity, respectively. An illustration of this coordinate space colored by each hierarchical cluster is depicted in Fig. 1A, along with summary points further describing the key physical characteristics of each group. These clusters include two rainfall-dominated groups (blue/light blue), two snowfall-dominated groups (orange/red), and five intermediate clusters representing various mixed-phase precipitation structures, all spanning both low and high intensity storm systems.

**Fig. 2.**
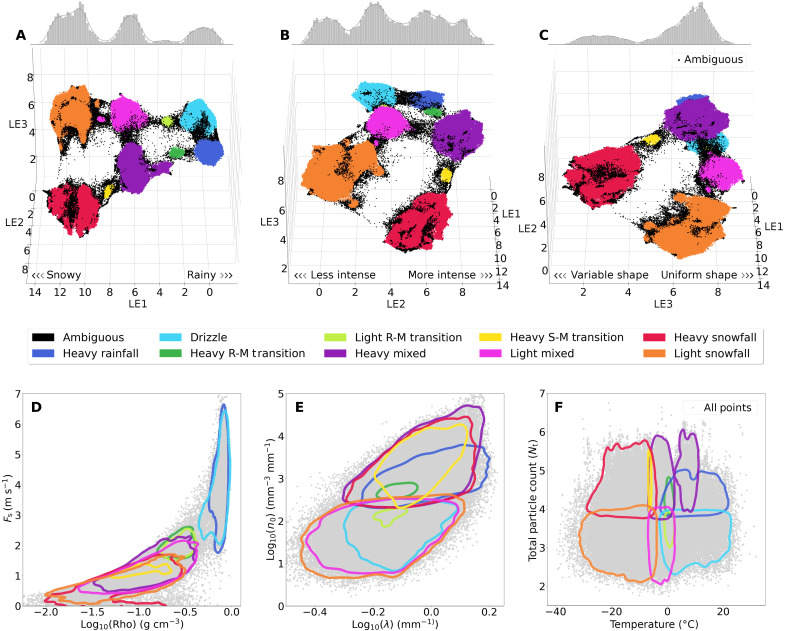
3D UMAP coordinate embeddings and physical variable space clusters. (**A** to **C**) UMAP coordinates for each latent embedding rotated along the LE1/LE2-axis plane, with a histogram and kernel density estimate (KDE) plot of nonambiguous point densities displayed along the top of each panel. (**D** to **F**) Precipitation cluster KDEs for each hierarchical cluster plotted in multiple physical variable spaces, including *F_s_*-log(Rho), Log(*n*_0_)-log(λ), and *N*_t_-*T*, where *F*_s_ is particle fall speed, Rho is the effective density, *n*_0_ is the PSD intercept, and λ is the PSD slope. All data points are plotted in gray behind each cluster KDE to visualize the underlying global particle distributions.

The respective size of each cluster in Fig. 1A varies substantially, with rainfall groups displaying tighter distributions of values near their centroids and similar circular shapes between intensity groups compared to snowfall, which displays wider distributions and more complex structures. From a physical perspective, this increase in complexity aligns with the more variable nature of regional snowfall processes, which exhibit greater diversity in particle sizes, shapes, and density compared to rain. These regional variations comprise the multiple, high-density subclusters within each snowfall group. Snowfall differences are further highlighted in the third embedded dimension (particle shape; LE3), which displays a wide separation between snowfall groups, indicating substantial differences in particle size and shape distributions (highlighted in Fig. 2). Regional subclusters are also present in the mixed-phase groups (specifically group 5; purple dashed region in Fig. 1A), highlighting unique regional processes such as orographic melt in the left purple arm of the cluster from the Olympic Mountains Experiment (*53*). Cases that do not map to a specific precipitation cluster are categorized as a 10th group (ambiguous). The distribution of these ambiguous cases typically forms distinct pathways between clusters, representing evolutionary periods during storms as particles change in temperature, density, intensity, or size/shape through processes such as melting, riming, aggregation, and sublimation. Ambiguous cases can also signify periods of extreme variability in the precipitation data during the 5-min interval; however, most appear to correspond with smooth, transitional pathways.

An example of such a transition event (heavy rainfall to mixed-phase precipitation to light intensity snowfall) is illustrated in Fig. 1B for a single 24-hour period at Marquette, Michigan. This event demonstrates how UMAP organizes the precipitation types on this day into five hierarchical groups, with each colored ring representing a discrete 5-min observation. As highlighted in Fig. 1C, the 2-m air temperatures ranged from +3° to +4°C until 17:00 UT, during which primarily intense, steady rainfall occurred, followed by less intense drizzle at 12:00 UT indicated by the lower intensity reflectivity values reported by the radar. At 17:00 UT, a cold front moves over the site, rapidly dropping surface air temperatures to −3°C within an hour. During this period, the hierarchical groups display a sudden jump to mixed-phase precipitation as the raindrops quickly became partially frozen. Temperatures continued to drop another 5°C over the remainder of the day, and particles continued to freeze and transition to the light intensity snowfall group as the storm weakened. Case study analyses indicate that while ambiguous-case transitional pathways are typically followed across individual events, jumps between clusters can occur when atmospheric conditions change quickly, and there are insufficient observations to smoothly move between groups (as we see from groups 2 to 5 at 17:00 UT).

Particle evolutionary pathways and the relative positions of hierarchical clusters are further highlighted in the 3D renderings of the UMAP coordinate space in Fig. 2 (A to C). In Fig. 2A, three distinct groups are apparent in the kernel density plot along the top axis. The three density peaks show precipitation phase separation in the UMAP coordinate space and represent (from left to right) cases of snowfall, mixed-phase precipitation, and rainfall, respectively. Examining these hierarchical clusters in physical variable spaces provides additional insight into their respective attributed precipitation types. For instance, in Fig. 2D, particle fall speed (*F*_s_) versus particle effective density (Rho) shows a distinct separation between rainfall, mixed-phase, and snowfall groups. Blue rainfall groups display high fall speeds (4 m s^−1^ or greater) with high effective density compared to the low fall speed and effective density of the orange and red snowfall groups. To enhance the visualization of the data, we rotate the LE1/LE2-axis plane by 90°. As a result, Fig. 2B now shows a clear left-right separation between low and high intensity groups (i.e., precipitation rate), respectively. In *n*_0_-λ space (Fig. 2E), high intensity groups are characterized by high PSD intercepts (*n*_0_) and steep slopes (λ). The steep slopes indicate a rapid decrease in the frequency of larger particles, leading to a shift in the PSD toward smaller median sizes (*54*). Conversely, low intensity groups exhibit shallower slopes, resulting in a relatively higher proportion of larger particles. Further, rainfall groups (blues) are more tightly coupled toward the center, while snowfall groups display wider distributions in intensity due to higher variability in regional snowfall processes.

In Fig. 2C, the particle shape embedding exhibits the weakest overall separation, featuring a bimodal distribution that separates the heavy snowfall group (red) from the other clusters. Notably, the centroids for the pink (light mixed), green (R-M transition), and blue (heavy rainfall and drizzle) regimes remain closely aligned along this dimension, suggesting that these regimes exhibit more uniform particle shapes. In general, raindrops typically display homogeneous morphologies. For the light intensity mixed-phase conditions, this uniformity likely arises as particles evolve in more stable temperature gradients, likely with moderate supercooled water, promoting slower, more consistent crystal growth. In contrast, the clear separation of the red (heavy snowfall), yellow (dendritic growth), orange (light intensity), and portions of the purple (mixed-phase) clusters implies that these colder and often more intense precipitation regimes are influenced by processes such as riming, collision-induced fragmentation, aggregation, and variable crystal growth, resulting in a broader diversity of particle habits. We therefore hypothesize that LE3 captures subtle variations in particle shape driven by the atmospheric conditions that produce different ice crystal types, as evidenced by the overlapping regions observed in Fig. 2D and their temperature relationships in Fig. 2F. Nonetheless, further analysis correlating this embedding with direct measures of particle complexity is needed to more conclusively validate this interpretation of LE3.

**Fig. 3.**
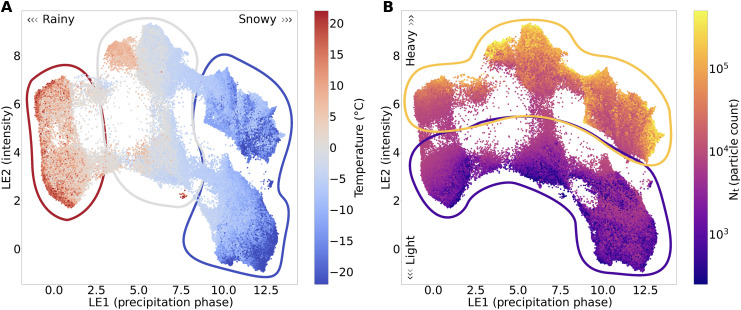
Physical variable distributions colored across the UMAP coordinate space. Embeddings colored by **(A)** 2-meter air temperature in °C with rainfall (red), mixed-phase (gray) and snowfall groups (blue); and **(B)** Total particle count with high intensity (orange) and low intensity (purple) groups.

Considering the precipitation phase (LE1) and storm intensity (LE2) embeddings, the UMAP coordinate space displays a smooth transition between physical variables associated with each embedding, indicating an overall preservation in the dataset’s global structure. The particle phase embedding, for instance, displays a smooth left-to-right temperature gradient between rainfall and snowfall groups (Fig. 3A). Mixed-phase groups in the center typically have temperature values ranging between −2° and +4°C, a range commonly associated with the transition between liquid and frozen precipitation over land (*55*). Similarly, the storm intensity embedding displays a smooth top-to-bottom gradient in total particle counts, a physical variable strongly associated with precipitation intensity (Fig. 3B).

Table 1 summarizes the classifications of the precipitation groups shown in Fig. 1, highlighting total observation days across all sites, the proportion of total cases observed at Marquette, and averages of key radar and meteorological variables at Marquette (as not all sites were equipped with the same surface radar instrumentation). These groups encompass diverse precipitation types, from heavy rainfall (40.1 days) characterized by warm temperatures (8.6°C) and intense near-surface reflectivity (31.6 dBZ) to light snowfall (160.6 days) with cold temperatures (−10.5°C) and low near-surface reflectivity (4.4 dBZ). Several distinct mixed-phase precipitation groups are also defined, capturing periods of slushy particles and sleet occurring near freezing temperatures.

**Table 1.** Overview of precipitation process hierarchical clusters. Attributes include total observation days across all sites, the proportion of total observations at Marquette (MQT) and associated mean summary variables at Marquette (*T* is temperature, *N*_t_ is total particle count, Ze is near-surface radar reflectivity, R is rain, S is snowfall, and M is mixed-phase). Total days are calculated over a span of 9 years.

ID	Cluster	Physical description	Total days	MQT (%)	*T* (°C)	*N*_t_	Ze (dBZ)
0	Ambiguous	Cases outside all defined UMAP hierarchical groups	66.1	46	−5.5	34,144	21.8
1	Heavy rainfall	Intense rainfall with numerous small drops	40.1	64	8.6	22,944	31.6
2	Drizzle	Light, steady rainfall	71.5	64	8.7	3,133	26.1
3	Heavy R-M transition	Intense sleet with dense ice pellets	3.2	47	0.1	25,833	18.3
4	Light R-M transition	Light sleet with dense ice pellets	3.2	60	0.5	3,512	12.7
5	Heavy mixed-phase	High volume of slushy, partially frozen particles	102.5	56	−2.0	71,112	15.6
6	Heavy S-M transition	Large snowflakes and aggregate particles	2.1	97	−5.8	70,766	15.3
7	Light mixed-phase	Low volume of slushy, partially frozen particles	64.7	58	−2.4	2,479	6.7
8	Heavy snowfall	Intense, heavy snowstorm	118.7	54	−11.6	45,354	11.2
9	Light snowfall	Light, fluffy snowfall	160.6	48	−10.5	2,684	4.4

### Comparing linear and nonlinear dimensionality reduction methods

To evaluate whether a nonlinear technique better represents precipitation types compared to a more interpretable linear dimensionality reduction method, we conducted parallel experiments with PCA using on the same dataset as UMAP. In previous work with a similar disdrometer dataset focused on snowfall, PCA efficiently separated the variable space into three primary EOFs: EOF1 represented snowfall intensity, EOF2 represented snow particle density, and EOF3 represented snowfall regime (*39*). However, PCA tended to produce overcrowding near the origin due to its preservation of linear relationships, leading to variance concentration along its principal components. In this study, using a higher-dimensional dataset with multiple precipitation types, we observed similar results using PCA. EOFs 1 to 3 represented physical features comparable to the embeddings identified by UMAP, but with a larger cluster of ambiguous cases toward the origin.

Applying HDBSCAN to the three PCA EOFs yielded two large hierarchical groups, corresponding to snowfall and rainfall. While potentially useful for basic rain-snow partitioning, this approach performs similarly to naive density threshold methods [e.g., selecting particles with an effective density above or below 0.4 (g cm^−3^) or a 0°C temperature threshold] and provides no information about mixed-phase precipitation, storm intensity, or particle size/shape. Following approaches similar to (*44*) and (*45*), we sorted cases with the most extreme EOF value combinations into discrete groups (i.e., manual clustering), as these map most strongly onto each of the PCA embeddings. This approach resulted in a more nuanced clustering of cases into six groups but increased the number of ambiguous cases located near the origin. Sensitivity tests found that using similar standard anomaly threshold values to both previous studies (i.e., a σ cutoff of 2 standard anomalies) provided a good balance by deriving groups that align with multiple, unique precipitation types while minimizing ambiguous classifications.

**Fig. 4.**
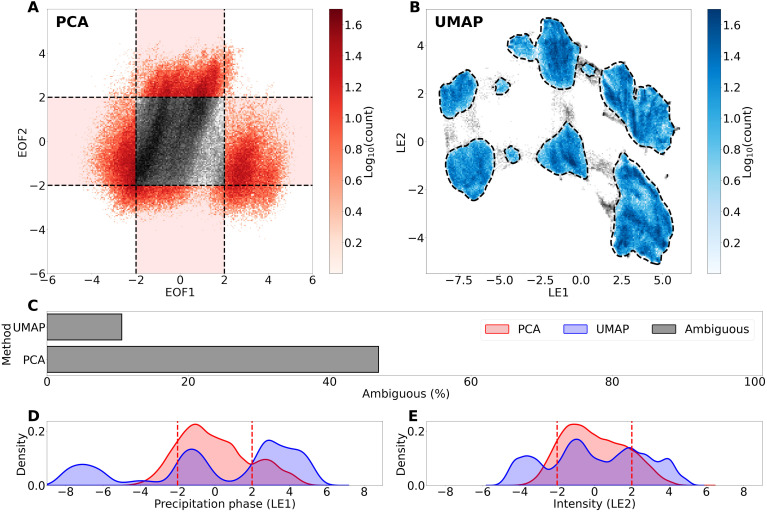
UMAP and PCA embedding distribution comparisons. (**A**) 2D joint distribution of the PCA precipitation phase embedding (EOF1) and storm intensity embedding (EOF2), with manual cluster thresholds in dashed black lines and ambiguous cases in gray. (**B**) Same as in (A) but for UMAP and HDBSCAN clusters outlined in dashed black lines. (**C**) Total ambiguous points for UMAP and PCA. (**D**) Precipitation phase embedding KDEs for PCA (red) and UMAP (blue), with dashed red lines showing the |2σ| cluster cutoff thresholds. (**E**) Same as in (D) but for precipitation intensity embeddings.

Ambiguous cases for the PCA and UMAP are illustrated in gray in Fig. 4 (A and B) with PCA cluster regions highlighted in the light red shaded regions. Note that Fig. 4A only displays EOF1/EOF2, so two additional clusters related to EOF3 along the *z* axis exist but are not visualized. Using this approach, PCA groups result in 36% more ambiguous cases than UMAP hierarchical groups (Fig. 4C). The silhouette score, a metric that measures cluster cohesion and separation, is also higher for UMAP groups (0.51) compared to PCA (0.37). This improved score (+0.14) suggests that the UMAP clusters are better matched to the global structure of the data, and cases are delineated from neighboring clusters. The kernel density estimate (KDE) distributions for analogous UMAP and PCA phase embeddings in Fig. 4D display a clear trimodality in phase for UMAP (blue) compared to the narrower, unimodal PCA distribution (red). A similar pattern is also observed for precipitation intensity separation in Fig. 4E.

### Precipitation cluster characteristics are consistent with independent observations

To further validate the physical precipitation types attributed to each UMAP hierarchical cluster, we analyzed independent ancillary observations from collocated vertically pointing radar measurements at Marquette. While other sites were also considered, not all had operating surface radars, and Marquette had the longest data record and widest range of observed precipitation types. We examine three case studies here, focusing on transition pathways between precipitation clusters related to each of the UMAP embeddings.

**Fig. 5.**
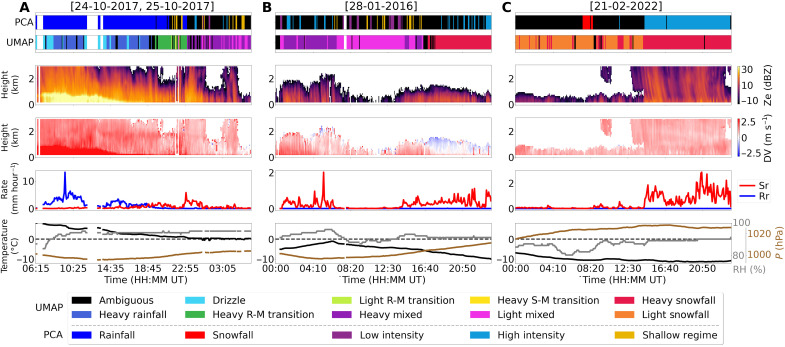
Case study comparisons of precipitation process clusters with ancillary observations. Comparisons include radar reflectivity (Ze) and Doppler velocity (DV) from the MRR where red indicates downward motion, surface snowfall (*S*_r_) and rainfall rates (*R*_r_) from the PIP, and surface meteorological observations [temperature (*T*), relative humidity (RH), and pressure (*P*)] from collocated weather gauges for three different 24-hour periods at Marquette. At the top of each column is a comparison of how each discrete 5-min interval was classified using UMAP and PCA. For PCA groups, deep blue is rain, red is snowfall, purple is low intensity precipitation, light blue is high intensity precipitation, yellow represents shallow regimes, and black is ambiguous. UMAP groups are the same as described in Fig. 1A. (**A**) 24-10-2017 to 25-10-2017 illustrates a prolonged mixed-phase precipitation event (highlighting LE1), beginning around 18:45 UT and lasting for 8 hours. (**B**) 28-01-2016 depicts a transition from a high-intensity convective mixed-phase precipitation event to less intense, shallow mixed-phase precipitation at 7:00 UT and finally to intense snowfall at 17:00 UT (highlighting both LE1/LE2). (**C**) 21-02-2022 displays the transition from a low-intensity shallow snowfall event to a high-intensity deep event with large aggregate particles at 13:00 UT (highlighting LE3).

The first case (Fig. 5A) was a prolonged mixed-phase precipitation event from 24 to 25 October 2017. From 06:00 to 17:00 UT, the event consisted primarily of heavy rain with large drops exceeding 2-mm effective diameter. This is indicated by an intense melting layer of high reflectivity values (>30 dBZ) below 1-km, high Doppler velocities (>3 m s^−1^), high surface rain rates, and warm temperatures (>5°C). From 17:00 to 23:00 UT, temperatures dropped from +3°C to just above 0°C, marking a multihour mixed-phase transition where raindrops began to partially freeze into heavy sleet, and nonzero rainfall and snowfall rates were both observed by the PIP. Between 23:00 and 07:00 UT the following day, temperatures remained around 0°C, and the PIP continued to report a mix of rain and snow. These transition groups were automatically identified in the UMAP hierarchical groups (highlighted by the colored bars at the top of Fig. 5A), showing clear shifts in precipitation type that aligned with the independent radar measurements. While the PCA groups effectively separated rain (deep blue bars) and some shallow snowfall (yellow bars), they display more ambiguity (black bars) and less continuity compared to the UMAP-derived groups.

The next event (Fig. 5B) was an extended mixed-phase and snowfall intensity transition on 28 January 2016. From 00:00 and 06:00 UT, a high-intensity shallow convective mixed-phase storm with dense, partially melted snow particles passed over the site. Temperatures ranged from −4° to −1°C, with medium-to-high Doppler velocities (>1.5 m s^−1^). As pressure climbed (to 1010 hPa) and temperatures fell (to −5°C) until 16:00 UT, reflectivity values also decreased, and UMAP groups classified this period as light intensity mixed-phase precipitation. Later in the day, as temperatures decreased further to −10°C, the precipitation type transitioned into the final high intensity snowfall group (red), denoted by a decrease in Doppler velocities, medium-intensity reflectivity values, and elevated snowfall rates above 0.5 mm hour^−1^. Although the PCA groups capture some of this intensity shift, they are notably sparser.

The third event (Fig. 5C) was a transition from a shallow lake effect snowfall to a deep convective snowstorm with record-breaking snowfall amounts on 21 February 2022, influencing the particle size and shape due to the change in depth of the system. Temperatures remained near −10°C for most of the day, with low reflectivity values observed from 00:00 UT to 13:00 UT, as a shallow snowfall system passed over the site. During this period, the PIP reported consistent small amounts of snowfall (<0.25 mm hour^−1^) and associated small ice particles. At 13:00 UT, the weather shifted to a deeper convective system with intense reflectivity streaks (>20 dBZ), larger particles (>2 mm), more stable relative humidity at 90%, and increased surface snowfall rates (some periods spiking above 2 mm hour^−1^). Both PCA and UMAP groups detected this snowfall regime shift, but the manual PCA technique marked most of the shallow event as ambiguous, while the UMAP hierarchical clusters provided a clearer mapping of both shallow and deep events.

### Long-term patterns reinforce physical precipitation cluster attributions

**Fig. 6.**
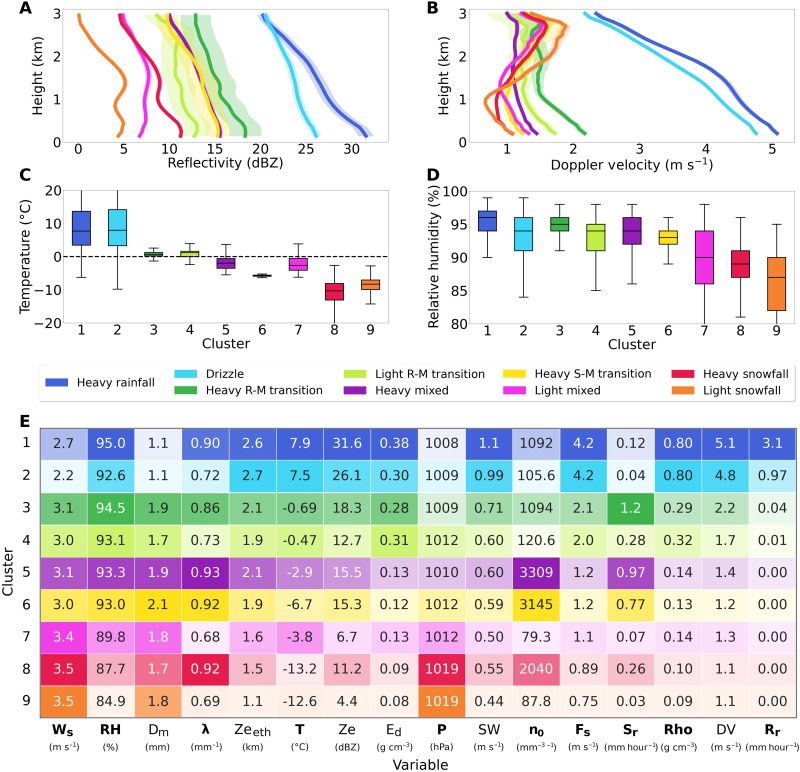
Long-term UMAP hierarchical cluster averages and variable comparison heatmap at Marquette. Mean vertical profiles of (**A**) reflectivity and (**B**) Doppler velocity from the MRR, with shaded regions indicating 95% confidence intervals. Box plot distributions of (**C**) 2-m air temperature (°C) and (**D**) relative humidity (%) for each cluster. (**E**) Mean value heatmap of disdrometer measurements, surface meteorological observations, and radar measurements across 9 years at Marquette, colored by their respective UMAP cluster and sorted by their cluster number. Deeper colors indicate higher relative values for each variable, with bolded variable names along the bottom indicating input variables to the dimensionality reduction.

Case studies can provide valuable event-scale insights**;** however, they do not necessarily reflect statistically significant long-term behaviors. To gain a deeper insight into the physical processes between hierarchical UMAP clusters, we therefore analyze 9 years of collocated radar measurements from a micro-rain radar (MRR) and surface meteorological observations at Marquette. We first derive mean vertical profiles (with 95% confidence intervals) of reflectivity and Doppler velocity up to 3 km for each UMAP group (Fig. 6, A and B). Notably, the blue rainfall groups (groups 1 and 2) show the highest reflectivity and Doppler velocities, as the high density of rainfall results in large power backscatter values and fast fall speeds (and therefore large Doppler velocities). In contrast, the snowfall-dominated groups (8 and 9, in orange and red) and the low-intensity mixed-phase group (7, magenta) exhibit the lowest mean reflectivity and Doppler velocity values due to their lower particle density and storm intensity. The remaining mixed-phase clusters fall between these two main groups, with relatively higher uncertainty due to their smaller sample sizes. Within these mixed-phase groups, the green rain-to-mixed transition groups, representing low and high intensity sleet, display higher fall speeds due to their increased density. The yellow snow-to-mixed transition group, likely representing dendritic snowflakes or aggregate particles, has high reflectivity from increased particle sizes but lower Doppler velocity due to their slower fall speeds from more complex shapes and low densities.

Temperature and relative humidity serve as inputs to UMAP, and boxplot comparisons of their hierarchical cluster distributions offer additional insights into the physical characteristics of each group, as shown in Fig. 6 (C and D). Rainfall groups display the widest temperature variability, with most cases well above 0°C (from 5° to 15°C) and only a handful of super cooled liquid water cases below 0°C. Transitional mixed-phase groups display much tighter distributions around 0°C, consistent with the narrow temperature range required for partial melting or freezing of particles. The snowfall groups (8 and 9) are much colder on average with wider distributions, highlighting the varied types of frozen particles (in shape, size, and density) formed under different atmospheric conditions and associated temperature ranges. The snow-to-mixed group 6 (yellow) has the narrowest distribution around −6.5°C, where cold surface conditions with high moisture availability likely contributes to the development of larger, intricate snowflakes. Relative humidity generally decreases from rainfall to mixed-phase to snowfall groups, with lower intensity groups showing slightly wider value distributions.

Figure 6E provides a summary of the relative differences between each UMAP hierarchical cluster for a variety of relevant environmental and microphysical variables. The rows represent individual UMAP groups (colored by their cluster value), and the columns represent various physical variables (bolded variable names are UMAP inputs). Interesting features include the fact that rain groups and those with denser or larger particles typically have higher mean reflectivity echo top heights (Ze_eth_) compared to snowfall groups. Group 6 (yellow), in particular, displays higher Ze_eth_ to other snowfall groups (and the highest mass-weighted mean diameter), indicative of particle growth and potential aggregation as the particle falls toward the surface. In addition, snowfall groups are generally associated with high surface pressure, low spectral width, and fast wind speeds. Notably, the snowfall rate for the low-intensity snowfall group is only marginally above zero, highlighting just how light and dry the precipitation in this group is compared to the denser, mixed-phase groups.

## DISCUSSION

This application of UMAP to a robust database of particle microphysical and collocated meteorologic observations yields three primary results. First, a 3D nonlinear manifold governed primarily by temperature, which determines phase, and by particle count, which determines intensity, spans the complete continuum from rain through mixed precipitation to snowfall. Second, connected pathways within this manifold connect nine novel latent microphysical regimes and accurately capture the complex spatiotemporal evolution of particles as seen in collocated observations. Third, because every regime is linked to specific particle characteristics, the manifold can be easily queried as a probabilistic parameter matrix (PM) that strengthens satellite precipitation retrievals and enhances weather and climate model parameterizations.

These results not only highlight the value of UMAP for organizing precipitation properties but also underscore its utility in revealing physically meaningful patterns across diverse meteorological conditions. By organizing this multidimensional dataset into a well-structured manifold, UMAP provides a clear delineation of complex, nonlinear processes such as mixed-phase precipitation transitional pathways and shifts in storm intensity. The general stability of the UMAP-derived coordinate embedding and the rainfall, mixed-phase, and snowfall clusters across all sites suggests that the derived manifold robustly captures the main features of global precipitation patterns and can generalize well to new observations. In addition, UMAP’s ability to capture nonlinear relationships facilitates a clearer and less ambiguous separation of precipitation process groups compared to traditional linear methods.

The UMAP-derived clusters offer tangible benefits for improving both remote sensing precipitation retrievals and numerical model parameterizations. The continued challenges in accurately partitioning precipitation phase, particularly within the critical −3° to +5°C range, underscore the critical need for novel, data-driven approaches. This clear separation of precipitation types shown here enables us to construct a robust PM for operational use. This PM serves as a robust a priori dataset for reducing error in space-borne retrievals, particularly in challenging mixed-phase regimes (*56*). As the primary source of observations in remote regions, improvements in spaceborne remote sensing retrievals would substantially enhance our understanding of changing precipitation patterns over the rapidly warming Arctic.

Furthermore, the clear delineation of mixed-phase transitional pathways and storm intensity shifts in the UMAP embedding provides critical insights into the nonlinear evolution of precipitation that is often oversimplified in conventional numerical models. By integrating the nonlinear cluster characteristics into a data-driven parameterization, this approach can enhance the bulk single moment microphysics schemes often used in numerical weather prediction models, improving constraints on precipitation type and rate and leading to more accurate simulations of surface hydrology and energy exchange processes (*57*). However, this study is limited by the available data, and further research is required to more conclusively identify rarer forms of precipitation such as hail by including observations from a broader range of disdrometers and locations [e.g., (*58*–*62*)]. Considering alternative PSD fits (e.g., a gamma distribution) beyond those examined here could also further refine the clusters to better capture cases of large aggregate particles and super-exponential distributions (*63*, *64*).

Despite these limitations, our findings underscore the utility of nonlinear dimensionality reduction for mapping subtle differences in the structure of regional precipitation. Moreover, beyond its previously discussed applications, this technique can be used to objectively identify extreme precipitation outlier cases along the edges of the coordinate embedding, providing valuable insights for enhancing weather prediction models and understanding rare meteorological events. The successful application of UMAP in this study demonstrates its power as a transformative tool in the broader atmospheric sciences, revealing physically meaningful nonlinear signals from complex global datasets and paving the way for more accurate predictions for weather climate applications in future studies.

## MATERIALS AND METHODS

### Experimental design

**Fig. 7.**
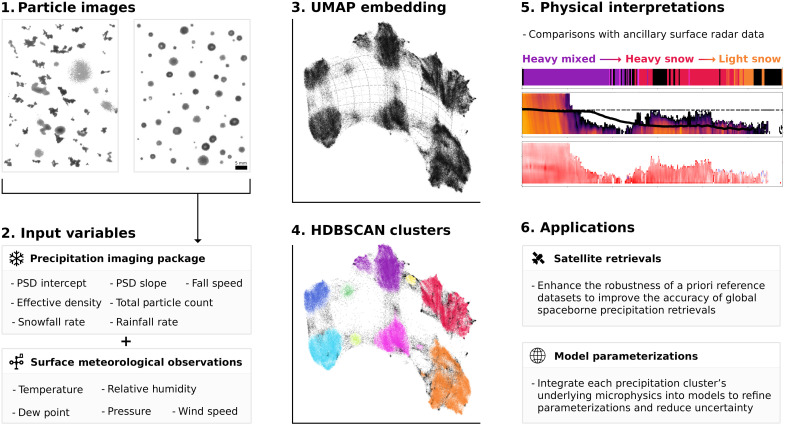
Project overview diagram. Schematic of (1) composite images of solid and mixed-phase/liquid precipitation particles observed by the PIP; (2) derived input variables from the particle images, including PSDs, fall speeds, and densities, accompanied by collocated surface meteorological observations describing the atmospheric conditions at the time of observation; (3) a visualization of the UMAP embedding coordinate space; (4) colored clusters derived from HDBSCAN that represent the primary modes of precipitation encoded in the variables from (2); (5) an example comparison between the HDBSCAN clusters and independent surface radar data (reflectivity and Doppler velocity from an MRR) to evaluate the accuracy of the assigned physical properties for each group; and (6) operational applications in fields such as spaceborne remote sensing of precipitation and global precipitation modeling improvements through an enhanced understanding of the key physical properties of different precipitation types.

We construct a dataset consisting of 128,233 5-min intervals of microphysical precipitation measurements and surface meteorological observations, gathered from seven sites over a 9-year period. To analyze this dataset, we apply UMAP to reduce its dimensionality from 12 variables to a 3D manifold that preserves the global relationships among variables. We use HDBSCAN to identify distinct clusters, capturing key precipitation types and particle evolution pathways, such as rain, snow, and mixed-phase transitions. To assess the effectiveness of UMAP, we compare it to PCA using the same dataset, evaluating how well each technique separates the underlying precipitation and surface meteorology characteristics that correspond to distinct precipitation phases and storm intensities and their ability to quantify the size of the ambiguous cluster. We further validate the physical relevance of the UMAP-derived clusters through a case study analysis, tracking how transitions between precipitation types, intensities, and storm regimes correspond to key atmospheric changes over 24-hour periods. Last, we examine long-term trends across the 9-year dataset to uncover the underlying modes of variability in precipitation processes across varying regional climates. This process is summarized in Fig. 7.

### Input dataset

Particle microphysical and collocated surface meteorological observations for this study are summarized in Table 2 and described in detail in (*43*). Microphysical measurements were obtained using the PIP, a high-speed video camera (380 frames per second) that captures images of falling particles and derives various microphysical properties from these images (*61*, *65*). These observations include distributions of particle size, vertical velocity, and effective density, as well as higher-order products aggregated over 1-min time intervals such as volume-weighted equivalent particle densities, liquid equivalent snowfall rates, and rainfall rate estimates (*65*–*67*). Additional surface measurements were collected from nearby weather stations including 2-m air temperature, relative humidity, pressure, wind speed, and wind direction. This dataset includes 1,070,000 min of precipitating observations of rain, snow, and mixed-phase particles. For this study, 128,233 5-min periods of particle microphysical attributes and collocated surface meteorological data were used. Sites were included if they had at least 1000 5-min periods and available collocated surface observations. Sensitivity tests with balanced-sample subsets from different sites yielded coordinate spaces similar in size and structure to those constructed using the full dataset.

**Table 2.** Overview of input variables. Summary descriptions of the dimensionality reduction input variable names, units, and their observational source.

Variable name	ID	Units	Log-scaled	Source
PSD intercept	*n*_0_	mm^−3^ mm^−1^	Yes	PIP level 3
PSD slope	λ	mm^−1^	Yes	PIP level 3
Fall speed	*F*_s_	m s^−1^	No	PIP level 3
Effective density	Rho	g cm^−3^	Yes	PIP level 3
Total particle count	*N*_t_	–	Yes	PIP level 3
Snowfall rate	*S*_r_	mm hour^−1^	Yes	PIP level 4
Rainfall rate	*R*_r_	mm hour^−1^	Yes	PIP level 4
Surface temperature	*T*	°C	No	Surface weather station
Relative humidity	RH	%	No	Surface weather station
Dew point	DP	°C	No	Surface weather station
Pressure	*P*	hPa	No	Surface weather station
Wind speed	*W*_s_	m s^−1^	No	Surface weather station

### Measurement sites

**Fig. 8.**
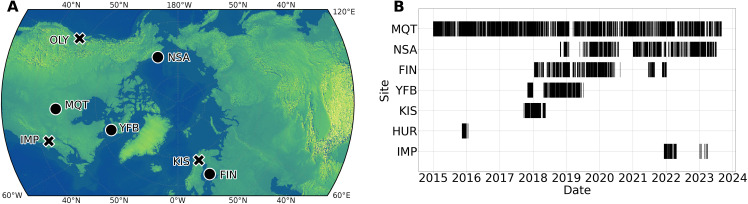
Measurement locations and observational temporal coverage. (**A**) Locations of each measurement site with long-term installations drawn in black circles and limited field campaigns in black X’s and (**B**) timeline of observational periods for each measurement site.

Microphysical and surface meteorological observations were collected from seven sites across five countries, spanning 37°N to 71°N (Fig. 8A). The dataset spans 9 years, from 14 January 2014 to 31 August 2023, and includes both limited field campaigns that lasted only a few months and long-term installations operating for the entire period (Fig. 8B). The specific study sites include MQT (Marquette, Michigan), NSA (North Slope, Alaska), YFB (Iqaluit, Canada), FIN (Hyytiälä, Finland), OLY (Olympic Peninsula, Washington State), IMP (Storrs, Connecticut), and KIS (Kiruna, Sweden). For a list of all measurement locations, their associated campaign names and references, and additional site-specific details [see section 2 of (*43*)].

### Ancillary observations

Collocated vertical pointing radar observations of reflectivity (Ze, dBZ), Doppler velocity (DV, m s^−1^), and spectral width (SW, m s^−1^) from nearby METEK MRRs were also collected and aligned with PIP observation periods. The MRR, a 24-GHz (K-band) Doppler radar, provides information about precipitation up to 3 km with vertical bin widths of 100 m (*68*). Raw power spectra are optimized for precipitation using details highlighted in section 2b of (*69*). Radar data were not used during model training; instead, it was set aside for independent validation of the precipitation type classifications derived from the dimensionality reduction techniques.

### Data preprocessing

The inputs used for fitting the dimensionality reduction models include the PSD intercept (*n*_0_, mm^−3^ mm^−1^), PSD slope (λ, mm^−1^), particle fall speed (*F*_s_, m s^−1^), effective density [the mass per unit volume across particles (Rho, g cm^−3^)], snowfall rate (*S*_r_, mm hour^−1^), rainfall rate (*R*_r_, mm hour^−1^), total particle count (*N*_t_), 2-m air temperature (*T*, °C), relative humidity (RH, %), dew point temperature (DP, °C), pressure (*P*, hPa), and wind speed (*W*_s_, m s^−1^) (Table 2). An inverse exponential fit (Eq. 1) was used to represent the PSDs for each 5-min period (*43*, *70*, *71*, *65*). The gamma function (Eq. 2) was also considered to capture distributions with longer tails due to the presence of large aggregate particles (*72*, *73*, *64*); however, fits converged to an inverse exponential in most cases, likely due to the dominance of snowfall observations with smaller particles less than 2 mm in diameter at most sites. To better understand the potential impact of observational error in the PIP or MET measurements, a series of sensitivity tests were performed, where 1, 3, and 5% Gaussian noise was added to all input variables before fitting; however, the UMAP manifold was robust in its shape and extent across all scenarios.

Before applying the dimensionality reduction techniques, we considered that the variables *n*_0_, λ, Rho, *S*_r_, *R*_r_, and *N*_t_ are hard bounded at zero and span several orders of magnitude, resulting in highly right-skewed distributions. To reduce this skewness and approximate normality, we applied a logarithmic scaling to these variables. Subsequently, all data were then normalized by subtracting the mean and dividing by the SD. This normalization helps maintain a physically meaningful distance metric when generating the UMAP manifold by accounting for the difference in scales of the inputs (*42*). Rows with missing or erroneous values were removed. In addition, only cases with wind speeds less than 7 m s^−1^ (25 km hour^−1^) were considered, as higher wind speeds can lead to issues with blowing snow, influencing the accuracy of the PIP microphysical observations (*74*, *75*)

$$n\left(D\right)=n_{0}\;e^{-\mathrm{\lambda D}}$$ (1)

$$\;n\left(D\right)=n_{0}\;D^{u}\;e^{-\mathrm{\lambda D}}$$ (2)

### Linear dimensionality reduction

PCA was implemented using the scikit-learn decomposition method (*76*). This technique performs a linear decomposition of the inputs using singular value decomposition to project them to lower-dimensional mappings. Specifically, PCA computes a covariance matrix of all inputs and then calculates eigenvectors and eigenvalues to identify the EOFs of the dataset (*41*). These EOFs represent directions of maximum variance in the dataset, thereby reducing the overall dimensionality and simplifying relationships between highly correlated inputs. PCA’s computational complexity is, $O\left(M*N^{2}\right)$ where *M* is the number of dimensions and *N* is the number of samples. Applying PCA to all 128,233 × 12 inputs results in eight total EOFs, accounting for 95% of the dataset’s variability. For this analysis, we selected the first three EOFs, as they represent 66% of the total variability, with each subsequent EOF representing ~5% or less. Because of the PCA coordinate space forming one large clump of points with little separation, techniques such as k-means or density-based clustering did not provide clear distinctions between varied precipitation types. Instead, clusters of points were selected using a methodology similar to (*44*) and (*45*), where a σ cutoff of two standard anomalies for each EOF was applied through a sensitivity analysis to partition the joint 3D distribution of EOF1, EOF2, and EOF3 into six equal-volume chunks. Using the |σ| = 2 threshold, we selected the points that most strongly mapped to each EOF for follow-up cluster analysis, allowing for a clearer physical interpretation of each PCA group.

### Nonlinear dimensionality reduction

We examined several nonlinear manifold-based dimensionality reduction techniques on the same dataset used for PCA, including Isomap, t-stochastic neighbor embedding, variational autoencoders, and UMAP (*77*, *78*, *42*). After evaluating the separation of points in the 3D embeddings of each technique, UMAP demonstrated the best computational efficiency and clearest separation of points into distinct groups, with embeddings displaying the most physically consistent properties, when compared to independent observations. Each of these techniques displayed an underlying consistency in their respective latent embeddings, revealing similar structures in the data despite differences in local organization and class separability. This suggests that the fundamental physical relationships captured by PCA are preserved across nonlinear techniques, although UMAP’s ability to better maintain both the local and global structure makes it the most suitable choice for this analysis. Furthermore, the UMAP embedding displayed a general stability when fit using different subsets of the data or even when entire sites were omitted from the training set, suggesting that new data would also likely map closely to the underlying manifold.

UMAP’s computational complexity is approximately *O*(*N*^2^), where *N* is the number of samples. UMAP constructs a high-dimensional, fuzzy topological representation of the input data by approximating the underlying manifold using a nearest-neighbor graph. This is achieved with nearest-neighbor descent for k-neighbor graph construction and associated approximate nearest neighbor search from (*79*). It then optimizes a low-dimensional graph layout by minimizing the cross-entropy between the fuzzy topological representations of the high-dimensional and low-dimensional data. This process was found to preserve both local neighborhood relationships and the global structure of the data, resulting in a low-dimensional manifold that effectively groups together points representing similar physical precipitating processes. However, note that, due to the nonparametric and stochastic nature of UMAP, the resulting embeddings may vary slightly between fits, necessitating careful interpretation of minor differences in the lower-dimensional representations.

HDBSCAN was then applied to objectively identify precipitation clusters (*80*). HDBSCAN constructs a hierarchy of clusters and extracts a flat clustering based on group stability, effectively identifying clusters of varying density with a robustness to observational noise. While using UMAP as a preprocessing step for HDBSCAN clustering is common, it is important to recognize that UMAP does not always preserve cluster density and can create artificial separations, introducing some uncertainty (*42*). Therefore, a post hoc evaluation of the clusters is essential to determine whether they represent genuine, physically meaningful groups.

### Hyperparameters

A grid search hyperparameter sweep was also performed to identify optimal values for the separation of points into distinct clusters using UMAP and HDBSCAN. For UMAP, default values were used except for *n_neighbors* = 750, *min_dist* = 0.22 and a weighted Manhattan distance metric, as these provided an optimal separation of precipitation types based on our input dataset. Using the same approach for HDBSCAN, we found that default values except for a *min_cluster_size* = 500 and *cluster_selection_epsilon* = 0.3 yielded the most physically meaningful clusters. While these values were found to provide the optimal separation of points into physically meaningful groups, the cluster architecture remained stable when *min_cluster_size* and *cluster_selection_epsilon* were varied around similar thresholds, suggesting that our results are not tightly coupled to a narrow set of hyperparameters.

### Applications

The derived PM, accessible through a simple function call, translates the resulting UMAP and HDBSCAN precipitation groups into a format for operational use. For instance, at each radar range gate, a Bayesian precipitation retrieval supplies temperature (*T*) and an estimate of total particle count (*N*_t_). A single query of the PM with these two inputs returns a 10 element probability vector *P* whose elements *P_k_* give the conditional probability that the gate belongs to latent microphysical regime *k*. Rather than selecting a single regime, we form a blended prior state vector *x*_a_ computed as the sum over *k* of *P_k_* μ*_k_*, where μ*_k_* is the cluster specific mean of all variables listed in Table 2. Because *P* changes continuously with *T* and *N*_t,_ the prior transitions naturally from primarily ice to primarily liquid as the atmospheric column warms, mitigating the need for subjective categorical thresholds between snow, mixed phase particles, and rain.

The same probability vector can refine bulk microphysical parameterizations in numerical weather prediction models in a similar manner. For instance, each precipitation cluster *k* carries a representative bulk particle density ρ*_k_* derived from the training data. Once per time step, the model queries the PM then updates the grid cell mean properties using weighted sums such as ρ equal to the sum over *k* of *P_k_* ρ*_k_.* These continuous weights allow the model to move smoothly from low density, mostly ice, particles to higher density, mostly liquid, particles in response to the evolving thermodynamic state of the atmosphere. With observations spanning seven diverse regional climates across nearly a decade, the resulting PM provides a stable and smoothly varying nonlinear mapping between precipitating particle phases that has been lacking in conventional priors and parameterizations.
