# Supplementary material for: Decoding global precipitation processes and particle evolution using unsupervised learning
Source: Sci Adv. 2025 Sep 19;11(38):eadu0162. doi: 10.1126/sciadv.adu0162 (PMC12448135; doi:10.1126/sciadv.adu0162)
Supplement: Supplementary file 1 — Supplementary Text Figs. S1 to S6 Table S1 [file sciadv.adu0162_sm.pdf]

Science Advances
AAAS

Supplementary Materials for

Decoding global precipitation processes and particle evolution using unsupervised learning

Fraser King *et al.*

Corresponding author: Fraser King, kingfr@umich.edu

Sci. Adv. **11**, eadu0162 (2025)
DOI: 10.1126/sciadv.adu0162

This PDF file includes:

Supplementary Text
Figs. S1 to S6
Table S1

Supplementary Text

The following are the supplementary results of additional sensitivity tests on the robustness of the Uniform Manifold Approximation and Projection (UMAP) fit, comparisons to other dimensionality reduction methods, and a visualization of the parameter matrix as a 3D histogram.

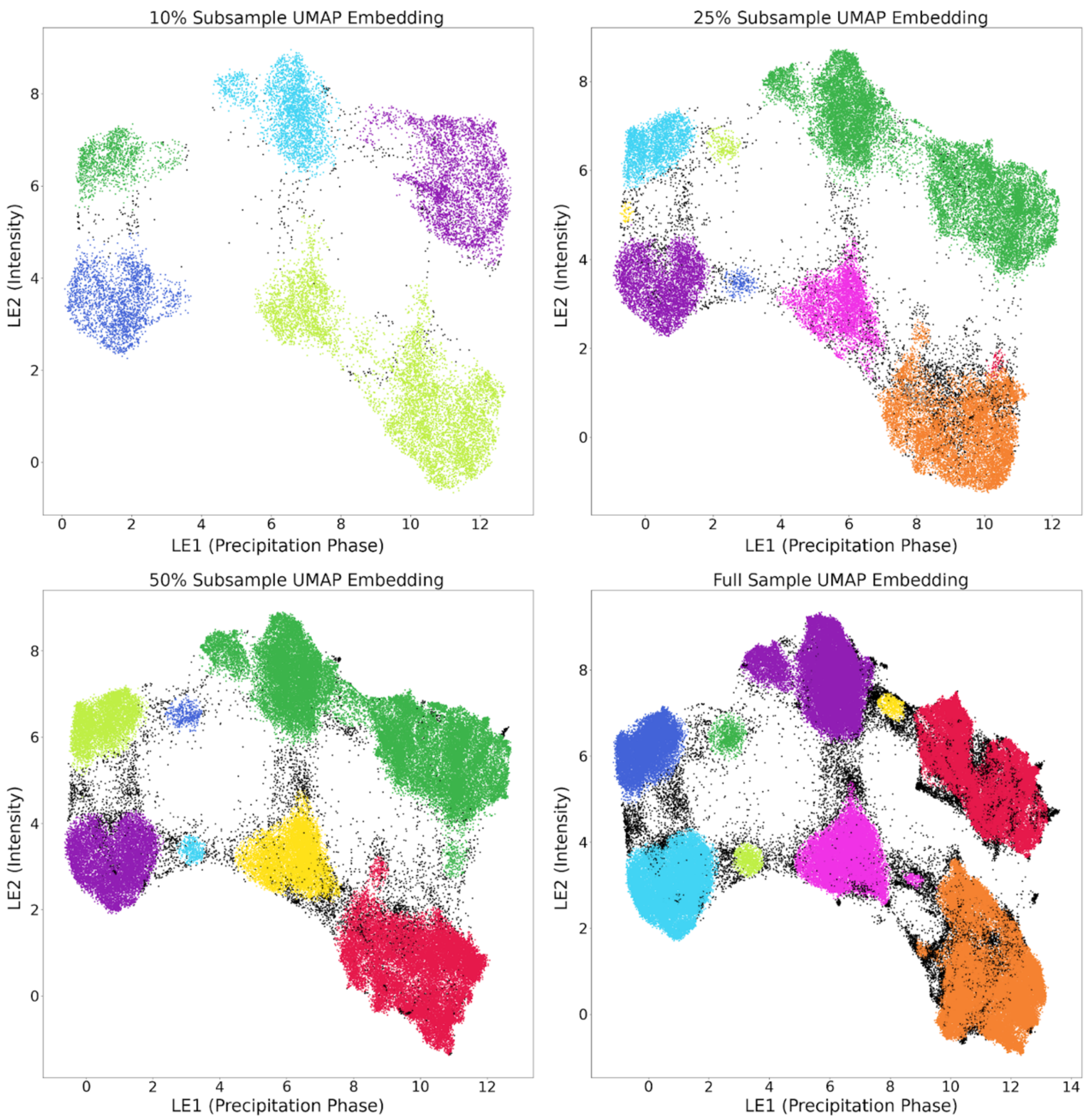

Fig. S1. 2-Dimensional UMAP embeddings using various randomly selected subsets of the data.

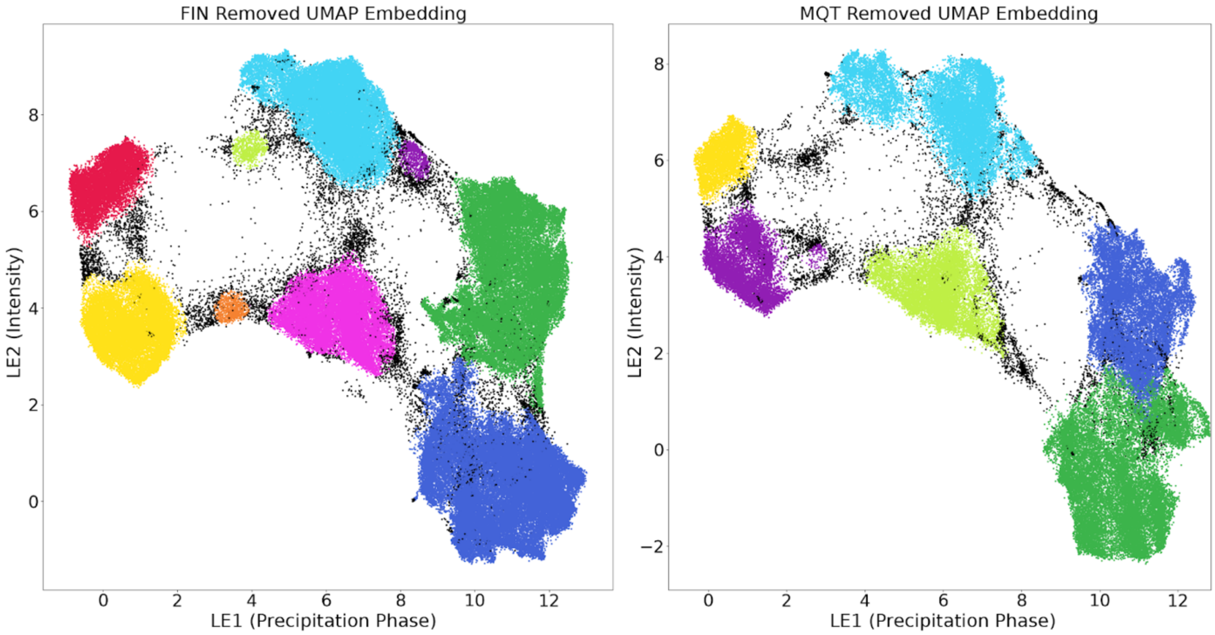

Fig. S2. 2-Dimensional UMAP embeddings fit with Finland (FIN) removed on the left, and Marquette (MQT) removed on the right.

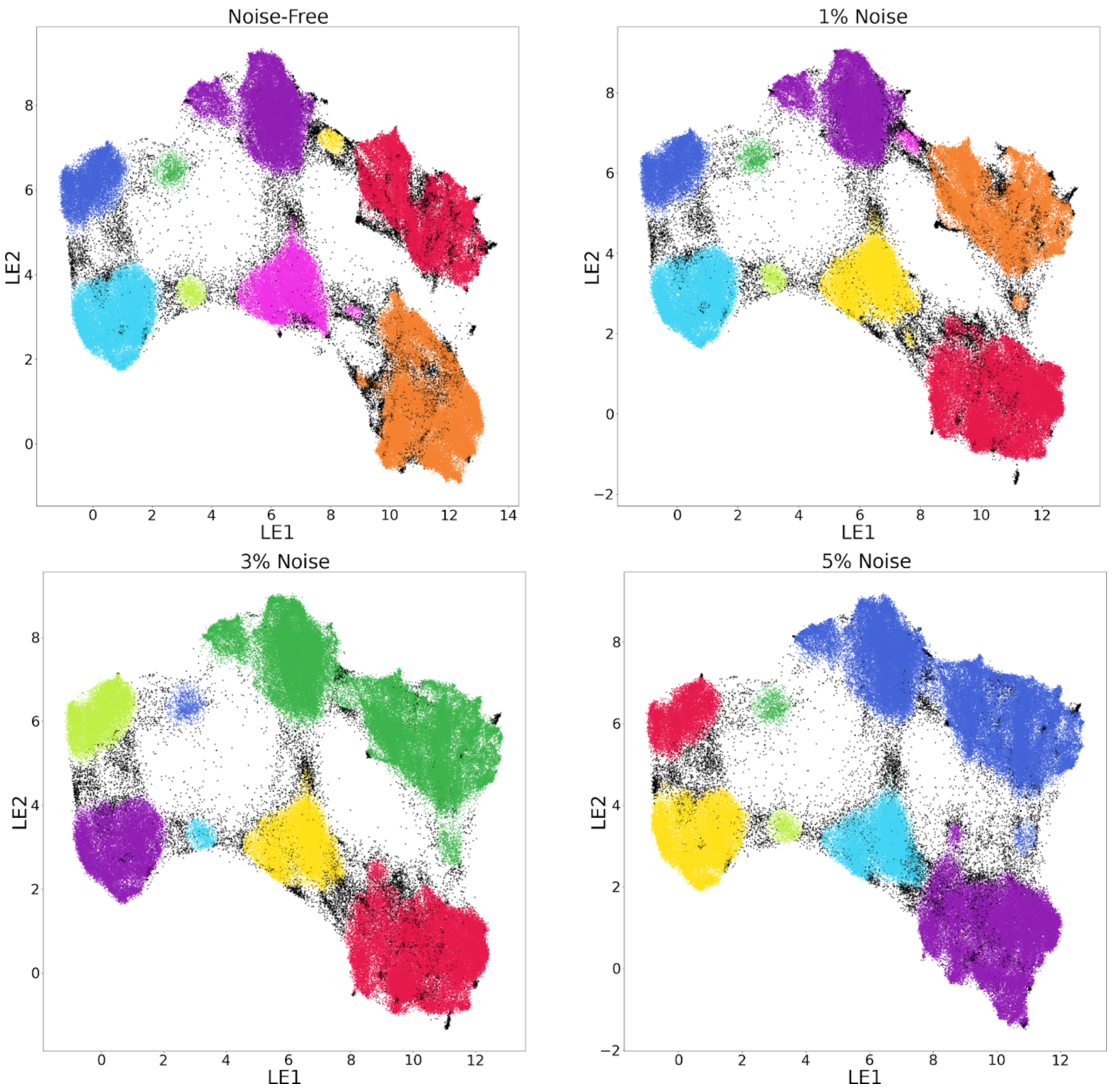

Fig. S3. Examples of different levels of Gaussian noise added to the input data before being fit using UMAP and HDBSCAN.

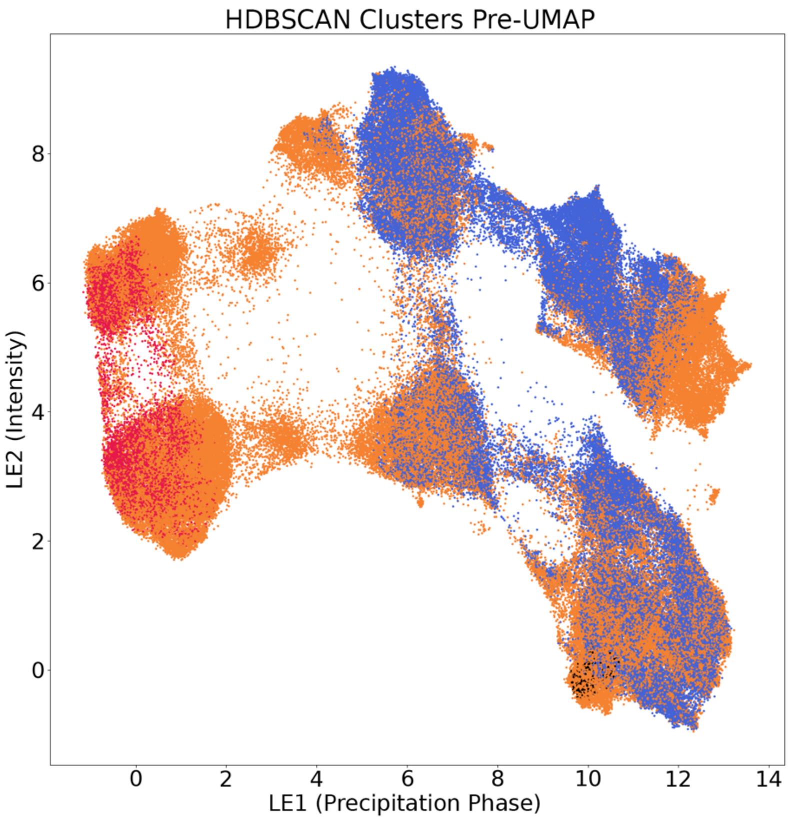

Fig. S4. Colored HDBSCAN clusters when applied to the raw 12-dimensional input and then projected into UMAP space.

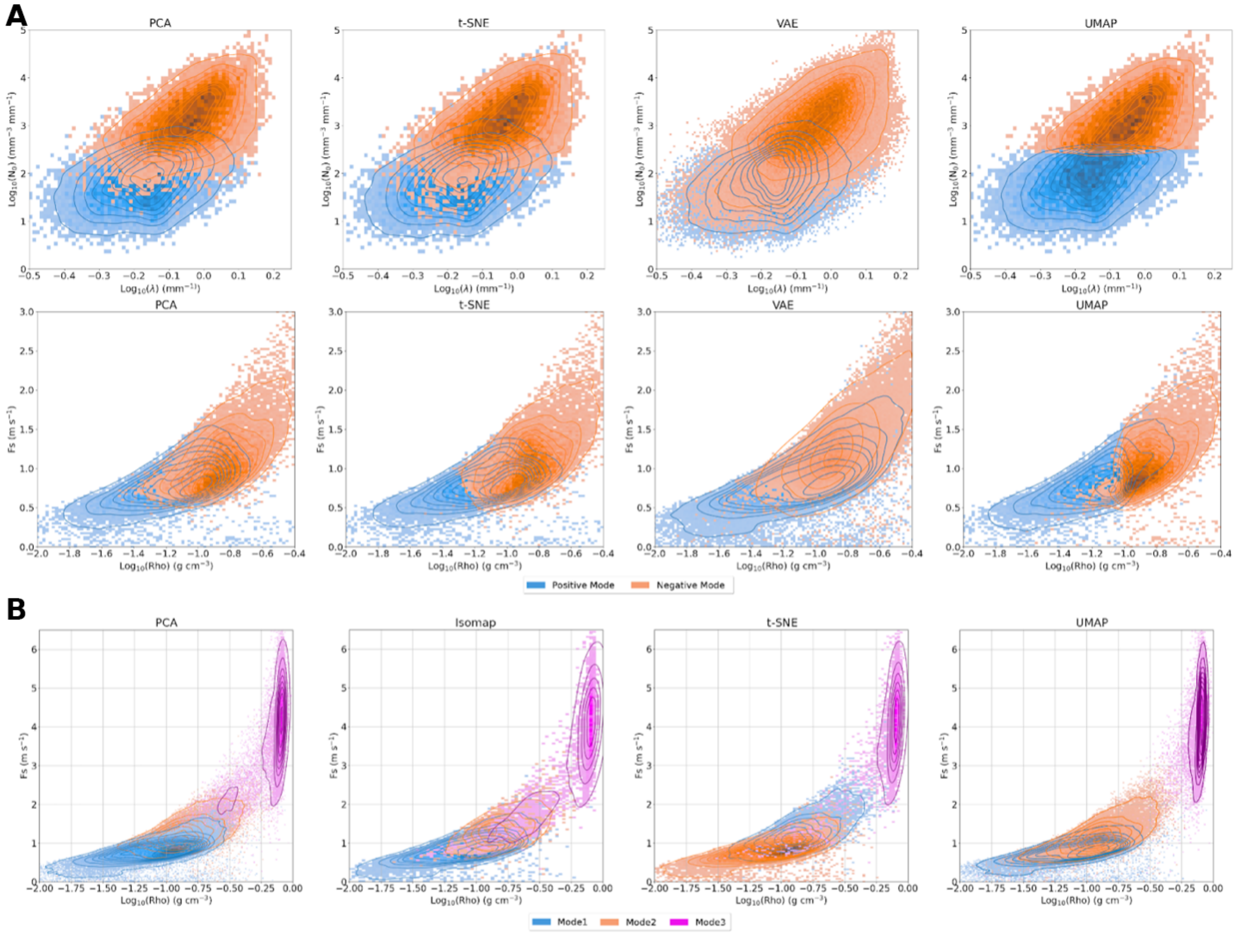

Fig. S5. (A) n_0-*lambda* space, and F_s-*Rho* space histograms overlaid with KDE curves showing the distribution of positive and negative modes of each dimensionality reduction's respective first (intensity) and second (phase) latent embeddings; **(B)** Similar to the second row in **(A)** but showing the distribution of multiple dimensionality reduction approaches separation of snow, mixed-phase and rain.

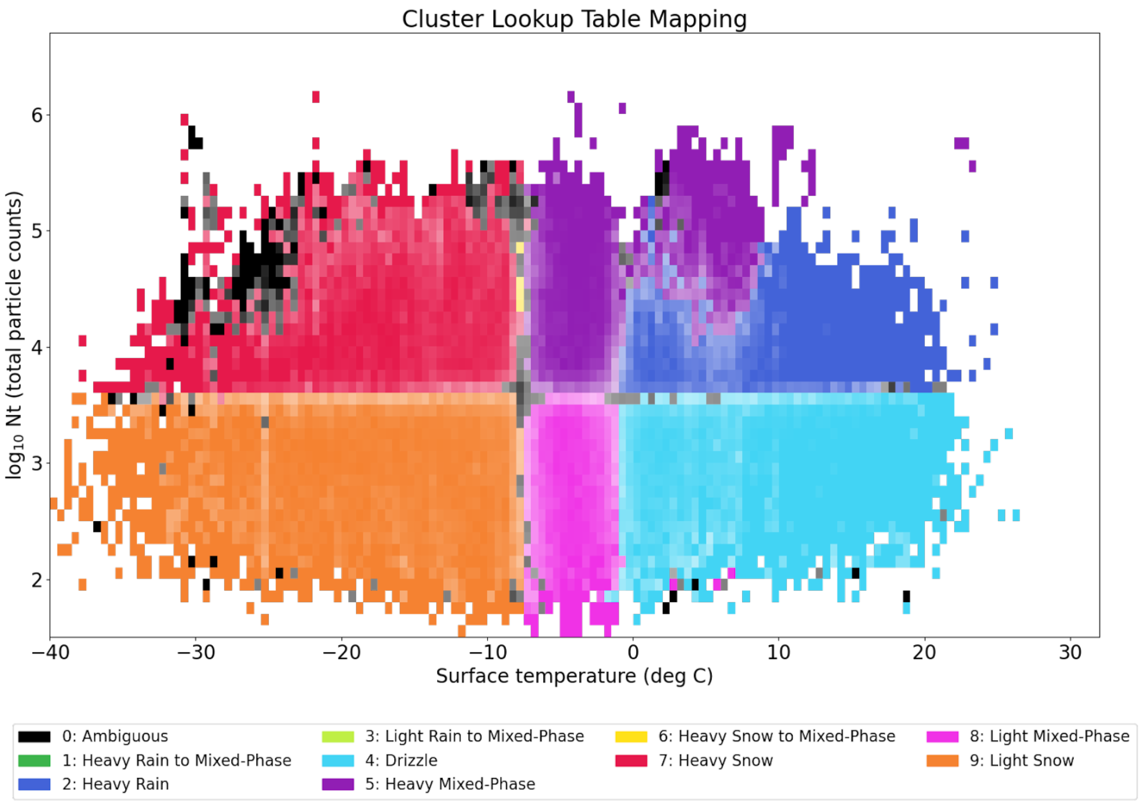

Fig. S6. UMAP+HDBSCAN 3D lookup table histogram showing which cluster is most closely aligned to each T-N_t bin, along with an alpha value corresponding to the probability of the fit for said bin (i.e., darker color means a higher probability for a specific bin while lighter colors are mixed between clusters).

Cluster	FIN	HUR	IMP	KIS	MQT	NSA	YFB
**0**	14.3	2.4	1.0	10.6	45.7	12.8	13.2
**1**	15.7	3.2	0.4	3.5	64.5	1.8	10.8
**2**	16.5	4.1	6.0	1.0	63.5	0.6	8.3
**3**	18.7	5.2	1.9	7.6	46.5	14.6	5.4
**4**	21.0	1.4	4.3	2.7	60.4	5.8	4.3
**5**	18.1	11.1	2.5	2.4	56.7	3.8	5.4
**6**	0	0	0	0.5	97.4	2.1	0
**7**	6.0	0	0.1	9.5	58.1	10.3	16.1
**8**	15.8	3.0	1.5	5.5	54.6	16.8	2.8
**9**	6.8	0	0.1	8.5	48.5	31.8	4.4

Table S1. Summary table of percentage occurrence of each UMAP+HDBSCAN cluster for each site over the full period. Note that cluster 0 is the ambiguous cluster.
